# Announcement: International Symposium of Pont-A-Mousson "Prospective Biology" Satellite Meeting: Vitamins and Biofactors General Aspects and Clinical Applications

**DOI:** 10.1155/MBD.1996.161

**Published:** 1996


					INTERNATIONAL SYMPOSIUM OF PONT-A-MOUSSON "PROSPECTIVE BIOLOGY"
SATELLITE MEETING:
VITAMINS AND BIOFACTORS
General aspects and clinical applications
Nancy, France, September 27 28, 1996
Organisers: J.-L. Guant & J.-P. Nicolas
Friday September.27, 1996
_9.00: Affival of the participants
First session: Epitheliums, mieronutrients and biofaetors
9.15: Y. Raoul: Ea suppl6mentation vitaminique
10.00: G. Sliek (Lille): L'assimilation du fer: physiologie et pathologie
10.30: Coffee
11.00: C. Roehette-Egly (Strasbourg)" R6tinoi'des, diff6rentiation 6pith61iale et cancer
12.00: Lunch
14.00: Posters
Second session: Status of micronutrients and risk populations"
cardiovascular diseases, AIDS and aging
14.30: B. Messing (.Paris): Micronutriments et SIDA
15.00" Oral presentations
16.30: Coff6e
17.00: Summa of the poster presentations
17.30: Jo Nve(Bruxelles): Le s616nium en pathologie vasculaire et du vieillissement
18.00: Co Jeandel (Nancy): Micronutriments et viedlissement
20.00" Gala dinner
Saturday Segtember 27, 1996
Third session" Micronutrients and biofactors:
actual biological evaluation methods
9.00: G. Le N4oel (Paris): Problbmes actuels de vitamines en biologie clinique
9.30: J. Van Wouwe (Delft): Biological diagnosis of zinc metabofism disorders in
ediat
0.00: Coffe
10.30" Oral presentations
12.00: D. Rosenblatt (Montreal)" Diagnosis of inherited disorders of folate and cobalamin
metabolism
12.30" Closing and lunch
Local Organisation Commitee: I. Aimone-Gastin, F. Belleville-Nabet, N. Danchin,
J.-L. Gu6ant, C. Jeandel, J.-P. Nicolas, M. Vidailhet, J.-M. Vignaud
Scientific Committee: C. Rochette-Egly, J.-L. Gu6ant, C. Jeandel, B. Messing, J. Nbve,
E. Nex, J.-P. Nicolas, D. Rosenblatt, G. Spick, J. Van Wouwe
Presidents: Y. Raoul, R. Gr/sbeck
Location:
Institut National Polytechnique de Lorraine (I.N.P.L.) Parking available
2, avenue de la For0t de Hay_e, F-54500 Vandoeuvre, France
Tel.: + 83 15 44 83 Fax: 83 15 35 91
Hotels"
H6tel Campanile, 1, avenue de la For& de Haye, F-54500 Vandoeuvre, France
Tel.: + 83 44 66 00 Fax" 83 44 66 17
H6tel Ibis, 2, all6e de Bourgogne, F-54500 Vandoeuvre, France
Tel.: + 83 44 55 77 Fax: 83 4 21 44
Travel"
* by plane: Nancy-Metz-Lorraine aiort and connec.tion to the symposium site by taxi
* by train: Nancy railway station and connection to tlae symposium site by bus (hne 4)
161

				

